# The Role of Docosahexaenoic Acid (DHA) in the Control of Obesity and Metabolic Derangements in Breast Cancer

**DOI:** 10.3390/ijms17040505

**Published:** 2016-04-05

**Authors:** Alessio Molfino, Maria Ida Amabile, Massimo Monti, Stefano Arcieri, Filippo Rossi Fanelli, Maurizio Muscaritoli

**Affiliations:** 1 Department of Clinical Medicine, Sapienza University of Rome, viale dell’Università 37, 00185 Rome, Italy; marida.amabile@gmail.com (M.I.A.); filippo.rossifanelli@uniroma1.it (F.R.F.); maurizio.muscaritoli@uniroma1.it (M.M.); 2 Department of Surgical Sciences, Sapienza University of Rome, viale Regina Margherita 324, 00161 Rome, Italy; massimo.monti@uniroma1.it (M.M.); stefano.arcieri@uniroma1.it (S.A.)

**Keywords:** docosahexaenoic acid (DHA), diet, food component, breast cancer, obesity, inflammation

## Abstract

Obesity represents a major under-recognized preventable risk factor for cancer development and recurrence, including breast cancer (BC). Healthy diet and correct lifestyle play crucial role for the treatment of obesity and for the prevention of BC. Obesity is significantly prevalent in western countries and it contributes to almost 50% of BC in older women. Mechanisms underlying obesity, such as inflammation and insulin resistance, are also involved in BC development. Fatty acids are among the most extensively studied dietary factors, whose changes appear to be closely related with BC risk. Alterations of specific ω-3 polyunsaturated fatty acids (PUFAs), particularly low basal docosahexaenoic acid (DHA) levels, appear to be important in increasing cancer risk and its relapse, influencing its progression and prognosis and affecting the response to treatments. On the other hand, DHA supplementation increases the response to anticancer therapies and reduces the undesired side effects of anticancer therapies. Experimental and clinical evidence shows that higher fish consumption or intake of DHA reduces BC cell growth and its relapse risk. Controversy exists on the potential anticancer effects of marine ω-3 PUFAs and especially DHA, and larger clinical trials appear mandatory to clarify these aspects. The present review article is aimed at exploring the capacity of DHA in controlling obesity-related inflammation and in reducing insulin resistance in BC development, progression, and response to therapies.

## 1. Introduction

### Relation between Obesity, Insulin-Resistance, Inflammation, and Breast Cancer Development

Research on modifiable risk factors for mortality in breast cancer (BC) [1], including the role of diet, has received increasing intensive attention by researchers worldwide [2], as they linearly affect the prognosis of the disease.

Obesity represents a major under-recognized preventable risk factor for cancer development and mortality and it increases cancer risk, including BC development [3]. Risk factors related to diet, particularly obesity, and low physical activity are often underestimated in assessing BC risk. The impact of obesity on the incidence and evolution of BC is not completely developed, due to multiple confounding factors, including biological differences of cancer subtypes [4]. During pre-menopause period, the subtype aggressive and estrogen receptor (ER) negative BC is more prevalent, whereas ER positive tumor subtypes are more frequent among post-menopausal women. Effects of obesity in postmenopausal women may be mediated by endogenous sex hormones (high levels of circulating estrogens and decreased levels of sex hormone binding globulin) [5,6]. This condition suggests that obesity is considered a relevant risk factor in post-menopausal BC. Plasma estrogen levels are increased in obese women and they are associated with almost a two-fold increased risk for BC development in postmenopausal phase. Moreover, aromatase expression is four to five times higher in BC tissue with respect to non-neoplastic areas of the same mammary gland [5]. In this light, estrogen concentrations of the mammary tissue resulted to be up to 50 times greater compared to plasma levels in healthy women in postmenopause. This condition has been demonstrated to be a crucial determinant in BC proliferation [7,8].

Instead, obesity in premenopause enhances the risk for developing the subtype of BC hormone receptor-negative [9,10].

This evidence supports that, independently from the hormonal derangements, obesity negatively influences BC risk, which is in part related to inflammation, insulin resistance and hyperinsulinemia related to obesity, and in part to hormonal interactions between cell types of the mammary gland [4].

Dietary polyunsaturated fatty acids (PUFAs) have been considered to play a key role in the pathophysiology of BC malignancy. Recent evidences [4,11] indicate that omega (ω)-3 PUFAs, including eicosapentaenoic acid (EPA) and docosahexaenoic acid (DHA), potentially reduce the circulating concentrations of eicosanoids produced by ω-6 PUFAs pathway [12] (Table 1). Moreover, the cytotoxic environment generated by ω-3 PUFAs has been shown to induce apoptosis and reduce cell proliferation in BC cells [12,13]. Long-chain ω-3 PUFAs showed high capability to sensitize BC cells to chemo- and radio-therapy, and thus potentially improve treatment efficacy, suggesting that ω-3 PUFAs oral intake, for which fish is the most important dietary source, might provide improvement of survival among women with BC [13].

Moreover, ω-3 PUFAs may be useful in a pan-anti-inflammatory approach to mitigate the inflammatory interactions of obesity on mammary tissue and subsequently to reduce tumorigenesis. It has been documented that increased intake of ω-3 PUFA, and especially of DHA, might reduce the incidence of BC associated to obesity. An American study found that higher intake of EPA and DHA from fish was associated with lower BC relapse and mortality [14].

High intake of ω-3 PUFAs is related to lower concentrations of proinflammatory biomarkers, *i.e.*, interleukin (IL)-1, IL-6, tumor necrosis factor (TNF)-α, C reactive protein (CRP), and to higher concentrations of anti-inflammatory cytokines, *i.e.*, IL-10 (Table 1) [11,15,16].

In addition, experimental data suggest a strong tumor-enhancing effect of ω-6 PUFAs and protective effects of ω-3 PUFAs on BC development [17]. Altering the balance between dietary ω-3 and ω-6 PUFAs has received attention as an approach for disease prevention, and tailored dietary intervention may be indicated as a promising tool to reduce BC risk [17].

The aim of the present article is to explore the role of DHA in BC development, progression and response to therapies. In particular, we focus on DHA capacity in controlling obesity-related inflammation, as well as insulin resistance in BC.

## 2. Relation between Overweight and Obesity during and after Treatment for BC and the Relative Risk of Recurrence

Obesity is a risk factor not only in influencing BC incidence, but also affecting poor clinical outcomes during and after therapies [18], and it is predictive of increased all-cause mortality [3]. Despite the higher survival rates in the last decades, BC survivors remain at considerable high risk of recurrence, as well as new primary BC, and the risk of mortality is higher compared to women without a prior diagnosis of BC [19]. Based on the available observational studies, patients who are overweight or obese at the diagnosis of BC, or who gain weight during therapies and follow-up, have a higher risk of relapse or mortality with respect to patients who do not increase their body weight after the diagnosis of BC [20,21,22]. Hormonal derangements, which are directly linked to the presence of obesity, and specifically the increased conversion of androgens to estrogens in peripheral adipose tissue, may play a crucial role in enhancing BC growth [1,23].

Studies documented that weight gain during BC treatments decreases the survival rate and increases the risk of recurrence [18,22,24].

Heideman *et al.* in a retrospective study conducted in Netherlands on patients treated for BC showed a significant increase in body weight occurred since the first year after the diagnosis. The highest gain of body weight in the five years after diagnosis was observed in patients who underwent combined systemic chemotherapy and endocrine therapy [24]. Rooney *et al.* confirmed that weight gain and changes in body composition are common during the first year after the diagnosis of BC [22], and that these conditions are associated with a poorer prognosis and poorer overall survival of BC patients [22]. Studies confirmed the association between BC and body weight gain for obese patients and, in particular, a risk of recurrence almost two times greater and a risk of death at 10-year follow-up greater with respect to patients maintaining usual body weight [18]. These data link obesity to BC mortality. In addition, weight gain contributes to the risk of comorbidities such as diabetes, cardiovascular disease, and depression [22].

The suggested mechanisms of weight gain after BC diagnosis and during cancer treatments include changes of the hormonal status, decreased physical activity, increased intakes, and low basal metabolic rate [21,23]. Increased circulating levels of leptin, insulin, and insulin-like growth factors (IGF-1) promote tumor cell proliferation, negatively affecting the prognosis of BC. In overweight and obese women the higher circulating levels of IGF-1 activate the phosphatidylinositol-3-kinases (PI3K)/Protein kinase B (Akt) pathway [24]. On the contrary, several studies indicate an increased overall survival in patients who are physically active after BC diagnosis [25,26], showing that physical activity and weight maintenance are associated with reduced BC recurrence and mortality [27]. An improvement in quality of life has been documented as a result of practicing aerobic exercise during and after therapies for BC. Physical activity improves mood and functional capacity, and reduces fatigue and nausea. Moreover, lifestyle interventions aimed at avoiding weight gain and increasing or maintaining a quite good level of physical activity, reduce BC incidence and ameliorate prognosis. These effects were associated with the modulation of inflammatory mediators, insulin, IGF-1 and estrogens [21].

## 3. Effect of ω-3 PUFAs and DHA on Inflammation in BC

Although hormonal modifications seem to play a driving role in breast carcinogenesis, the cytokines production and the consequent increased inflammatory status are recognized as clinically relevant features during BC growth and progression [28,29].

Chronic inflammation contributes to cancer growth and progression, playing a major role in the neoplastic process. Almost 20% of tumors are associated with inflammation [30]. Considering the elevated inflammatory state, which is present in obesity [31,32], the production of adipose tissue inflammatory mediators related to obesity could exacerbate tumorigenic effects associated with inflammation [4]. High body fat content determines insulin-resistance and increased circulating concentrations of insulin-like growth factor (IGF) and adipokines, including leptin and adiponectin, and abnormal values of TNF-α and IL-6 [33]. Insulin-like growth factor, IL-6, and leptin promote neoangiogenesis and trascriptional factors and increase aromatase activity determining production of estrogens [23]. It is known that leptin concentrations are directly proportional to total body fat [16] and high leptin levels are related with greater risk of BC growth and progression [34]. Most of these adipokines are upregulated in overweight and obese BC patients and modulate intracellular pathways influencing cell malignant transformation associated with inflammation [35].

**Figure 1:**
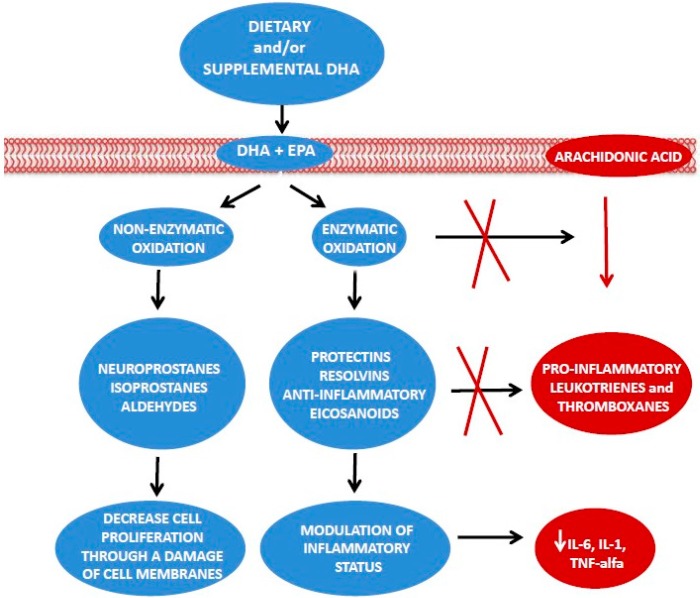
Enzymatic way and non-enzymatic way for ω-3 PUFAs. DHA, docosahexaenoic acid; EPA, eicosapentaenoic acid; IL-6, interleukin-6; IL-1, interleukin-1; TNF-alfa, tumour necrosis factor-α.

Observational studies linked inflammation to imbalance of dietary intake of ω-3 and ω-6 PUFAs [2] (Figure 1), and specifically a higher intake of ω-3 fatty acids is associated with lower levels of proinflammatory cytokines (IL-2, IL-6, TNF-α) and with higher anti-inflammatory patterns (IL-10, TGF-β) [16]. Imbalance of dietary intake of ω-3 and ω-6 PUFAs (also expressed as ω-6/ω-3 ratio) reached with a high ω-6 PUFAs diets is associated with increased adipokines levels, higher proinflammatory cytokines secretion, and hyperinsulinemia, which represent factors all contributing to chronic inflammation [1,23]. Dietary ω-6 PUFAs increase the eicosanoids synthesis, increasing inflammatory status and the immune response [2]. These events condition cell angiogenic and proliferation properties [2]. The ω-6/ω-3 ratio may be a stronger predictor of inflammation with respect to the fatty acid alone [16]. Alfano *et al.* [16] documented in a prospective study on more than 600 BC survivor women, a significant linear association between inflammatory markers, particularly CRP, and ω-6 and ω-3 intake. Women with the highest ω-6/ω-3 ratio who were not supplemented with ω-3, had the highest serum CRP levels, whereas women with the lowest ω-6/ω-3 ratio and who were assuming ω-3 supplements showed the lowest serum CRP levels [16].

In the recent years, western diets are significantly enriched in ω-6 PUFAs content, determining an imbalance in the ω-6/ω-3 PUFA ratio. Increased ω-6 PUFA contents and a high ω-6/ω-3 ratio up to a 16:1 ratio, have been indicated to favor the development of several diseases, including cardiovascular disease and different types of tumor, whereas higher ω-3 PUFAs concentrations have been shown to exert protective effects [36].

Experimental studies showed that dietary supplementation of ω-3 PUFAs was related to reduced adipose tissue inflammation and increased insulin sensitivity in obese mice [37], highlighting that ω-3 PUFAs might be useful in reducing obesity-associated inflammation, metabolic derangements during cancer [38], and tumorigenic risk [28,39]. Similarly, clinical studies conducted in overweight patients confirmed the anti-tumorigenic effects mediated by ω-3 PUFAs, showing that ω-3 PUFA supplementation up-regulated the expression of genes involved in cell cycle control [40].

In human clinical trials, dietary ω-3 PUFAs increased adiponectin levels in obese and overweight subjects, demonstrating the potential utility of ω-3 PUFA to restore adiponectin function and to stimulate its anti-inflammatory properties [4], and consequently the anti-tumorigenic effects of this adipokine [41].

Nevertheless, a randomized trial in healthy young adults, providing increasing doses of EPA + DHA up to 1.8 g/day for five months, found a marginal decrease in TNF-α levels and no change in IL-6 levels, documenting no effect of marine PUFAs on circulating inflammatory molecules [42]. However, we acknowledge that healthy adults are unlikely to have high levels of inflammatory markers, hence less likelihood of showing a response. In this light, we found several positive results, when considering the effects of omega-3 fatty acids administration on inflammation associated with several diseases [43], including obesity [44].

Also, it is known that the effects of ω-3 PUFAs, which are able to inhibit cancer development and progression, are related to the number of their skipped dienes [45]. Studies documented that the negative control of cancer cells proliferation was more evident during a treatment with DHA, than with alpha-linolenic acid or with EPA [46]. This indicates that PUFAs need a peroxidation process to become active [47]. Lipids peroxidation, which is followed by secondary metabolites transformation, represents an important pathway able to reduce cancer cell proliferation rate [48]. A synthesis of non-enzymatic metabolites, as neuroprostanes or isoprostanes [49], might explain the anti-proliferative action of DHA in BC (Figure 1). Neuroprostanes and isoprostanes are able to decrease cell growth through damaging cell membranes, by altering membrane composition and structure. These changes in cell membranes can inactivate its transport systems and/or enzymes activity [48,49]. In addition, metabolites of lipid peroxidation reduce cancer cell proliferation through the inactivation of DNA polymerase reactions, inducing intramolecular linkages between nucleic acids and proteins [49].

Further investigations will need to address the role of additional markers of inflammation, including cellular adhesion molecules, and of lipid peroxidation to better clarify the clinical effects of DHA supplementation on inflammatory status and cancer cell growth.

## 4. Effect of ω-3 PUFAs and DHA on Insulin-Resistance and Obesity in BC

**Figure 2:**
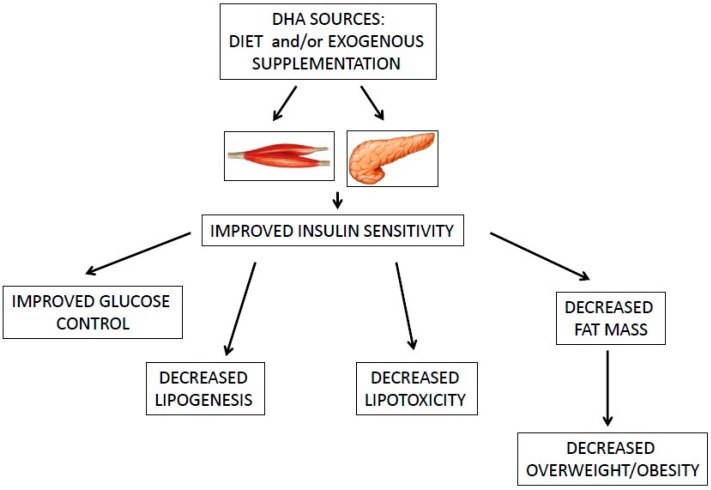
The role of DHA in insulin and glucose metabolism. DHA, docosahexaenoic acid.

Omega-3 PUFAs are considered to play a protective role on BC risk in obese women which might be related to increased uptake of fatty acids by the cells and to decreased inflammation and enhanced insulin sensitivity in adipose tissue [4] (Figure 2). Increased estrogen synthesis, as a consequence of higher aromatase expression in adipose tissue, is associated with increased obesity related BC risk [4]. High intake of ω-3 PUFAs might decrease endogenous estrogen production via inhibition of aromatase activity/expression [4]. However, this aspect needs to be better clarified in further studies.

Inverse associations between ω-3 PUFA dietetic intake and cancer risk have been also reported in Asian women having a dietary intake of marine PUFAs 40 times greater than women from Western countries, using validated questionnaires of dietary intakes [50,51,52]. In particular, Gago-Dominguez *et al.* administered a validated questionnaire on food frequency to Singapore Chinese women, consisting of more than 160 food and beverage items, to assess patient’s fatty acids intake and BC risk [50].

Chajès *et al.* conducted a case–control study in Mexico on 1000 patients affected by BC and over 1000 control healthy subjects to specifically investigate the relation between obesity, ω-3 PUFA intake and BC risk [53]. It was found that an increased risk of BC associated with greater ω-6 PUFA dietary intake in premenopause (*p* = 0.04) and, on the other hand, a decreased risk of BC associated with higher ω-3 PUFA intake in obese women (*p* = 0.008), but not in overweight nor in normal weight women. In particular, DHA intake in Mexican women was about 10 times lower than the one reported in Western populations [53].

Patterson *et al.* showed that in BC survivors the intake of marine PUFAs related to improved BC prognosis [14]. More than 3000 women diagnosed and treated for non-metastatic BC were considered, documenting as higher dietary intakes of EPA and DHA were correlated with lower BC recurrence and with a dose-dependent reduction of mortality risk in overweight women (average body mass index of 27.3 kg/m^2^). From the other side, DHA and EPA intakes from fish oil supplements were not associated with BC relapse or mortality [14].

MacLean *et al.* have reviewed the literature to assess the role of ω-3 PUFAs in preventing cancer [54] and found no overall trend across different cohorts and categories of ω-3 PUFA consumption, suggesting that low ω-3 PUFA levels might not be associated with cancer risk.

In contrast, a recent meta-analysis by Zheng *et al.* supports a protective role of marine derived ω-3 PUFA on the incidence of BC [55], confirming that ω-3 PUFA intake was significantly inversely associated with BC in postmenopausal but not in premenopausal women. This observation highlights also that specific benefit of marine ω-3 PUFA may be obtained usually after long-term exposure. This finding might be determined by a possible different role of premenopausal and postmenopausal adiposity in conditioning risk of BC. One recent meta-analysis suggested that high body mass index appears protective against premenopausal BC but it still represents a risk factor for postmenopausal BC [56]. A mechanistic explanation of these findings is still lacking and further studies are needed.

In a French study, including approximately 56,000 women, Thiebaut *et al.* investigated the relationship between individual PUFA intakes, evaluated by the administration of dietary history questionnaires, and BC risk and found no association [57].

The Women’s Healthy Eating and Living (WHEL) was a randomized trial conducted among more than 3000 BC survivor women, who were assigned to receive a phone call counseling program, supplemented with cooking classes participation and promotional newsletters about diet (intervention group), or to receive only print materials describing dietary guidelines (control group) [58]. Although the first group significantly increased fruit and vegetables intakes and reduced fat intakes, no differences in BC recurrence nor mortality were documented after a mean follow-up period of seven years [58].

Interpretation of the reported data is limited by differences regarding the characteristics of the populations studied and by differences in the methods used to assess exposure to ω-3 fatty acids and the risk of BC.

Monk *et al.* [4] provided findings that dietary ω-3 PUFAs, particularly marine-derived EPA and DHA, which present anti-inflammatory properties in obesity [41] and anti-neoplastic role in BC [59,60], may be an additional strategy in the prevention and treatment of obesity-related BC, by reducing inflammatory status within the mammary gland.

Omega-3 PUFAs have been demonstrated to target multiple aspects of the obese BC phenotype [4]. Nutritional interventions appear able to modulate treatment toxicity in different type of cancers [11,61]. In particular, DHA supplementation during chemotherapy enhanced the toxic effects of anti-mitotic therapies [59]. This effect was determined by the incorporation of DHA into membrane lipids, altering membrane permeability, and improving cellular drugs uptake [59]. In this view, a clinical study showed that DHA supplementation during chemotherapy increased survival in metastatic BC patients. The anti-cancer property of DHA may be determined by its ability to counteract cell growth and facilitate apoptosis [13]. In addition, Patterson *et al.* indicated that BC patients with greater intakes of EPA and DHA from food, but not from other sources such as supplements, showed a significant dose-dependent reduction in mortality from any cause [14].

Therefore, other studies, evaluating the role of dietary PUFAs on BC risk, found inconclusive epidemiological evidence [62]. Although several findings indicate that DHA does not present modulating effects on insulin sensitivity, it does not exclude the possibility that DHA provides a positive effect on insulin metabolism [63]. More importantly, obesity is a cause and consequence of inflammation which is a key determinant in the pathogenesis of insulin resistance. Understanding the influence of these modifiable risk factors in BC appears of high importance as they directly affect patients’ prognosis.

## 5. Conclusions

The prevention and treatment of BC represent highly relevant public health issues. These aspects are important in the research field especially regarding the relations between diet, lifestyle, and BC.

A possible preventative and therapeutic role of DHA in BC development and progression has been highlighted. Robust data indicate several anti-inflammatory effects of DHA suggesting that this ω-3 PUFA may represent a valid candidate for primary and secondary BC prevention in subjects at increased risk as well as BC survivors [64]. It remains uncertain whether such biological effects of DHA, consisting in altering inflammatory pathways, are clinically relevant at typical dietary doses.

Controversy exists regarding the potential anticancer effects of marine ω-3 PUFAs and especially DHA, and larger clinical trials appear necessary to clarify these aspects.

## Figures and Tables

**Table 1:** Docosahexaenoic acid (DHA) in breast cancer—Key points.

Research on modifiable risk factors in breast cancer is receiving clinical relevance because they affect the prognosis of the disease [1,2].
Data indicate that obesity increases breast cancer risk in part due to hormonal interactions and in part to inflammatory and insulin-resistance mechanisms [3,4,5,6].
Omega-3 fatty acids are metabolically active lipids with anti-inflammatory properties [13,14,15,16].
Omega-3 fatty acids and, in particular, DHA ameliorate obesity-induced inflammation and insulin-resistance [4,36,37].
Considering the role of inflammation in breast cancer, there is an increasing rationale for the use of DHA in combination with anticancer therapies [14,15,16,64].
Additional evidence is needed to assess the protective role of DHA breast cancer.
